# Comparison of Phenolic Compounds and the Antioxidant Activities of Fifteen *Chrysanthemum morifolium* Ramat cv. ‘Hangbaiju’ in China

**DOI:** 10.3390/antiox8080325

**Published:** 2019-08-20

**Authors:** Jinyan Gong, Bingquan Chu, Lingxiao Gong, Zhongxiang Fang, Xiaoxu Zhang, Shaoping Qiu, Jingjing Wang, Yali Xiang, Gongnian Xiao, Haina Yuan, Fuping Zheng

**Affiliations:** 1 Beijing Advanced Innovation Center for Food Nutrition and Human Health, Beijing Technology and Business University, Beijing 100048, China; 2 Zhejiang Provincial Key Lab for Biological and Chemical Processing Technologies of Farm Product, School of Biological and Chemical Engineering, Zhejiang University of Science and Technology, Hangzhou 310023, Zhejiang, China; 3 School of Agriculture and Food, The University of Melbourne, Parkville, VIC 3010, Australia; 4 College of Food Engineering and Biotechnology, Tianjin University of Science and Technology, Tianjin 300457, China

**Keywords:** chrysanthemum, HPLC, phenolic compounds, principal component analysis, antioxidant capacity

## Abstract

This study investigated the phenolic compounds of 15 *Chrysanthemum morifolium* Ramat cv. ‘Hangbaiju’, including 6 ‘Duoju’ and 9 ‘Taiju’, using high performance liquid chromatography (HPLC). The antioxidant activities of these ‘Hangbaiju’ were estimated by DPPH, ABTS and FRAP assays. Results show that a total of 14 phenolic compounds were detected in these flowers, including 3 mono-caffeoylquinic acids, 3 di-caffeoylquinic acids, 1 phenolic acid and 7 flavonoids. ‘Duoju’ and ‘Taiju’ possess different concentrations of phenolic compounds, and ‘Taiju’ exhibits higher caffeoylquinic acids and stronger antioxidant activities than ‘Duoju’. Caffeoylquinic acids show a strong correlation with the antioxidant activities of the samples. Principal component analysis (PCA) reveals an obvious separation between ‘Duoju’ and ‘Taiju’, using phenolic compounds as variables. Apigenin-7-*O*-glucoside, 3,5-di-*O*-caffeoylquinic acid, luteolin and acacetin were found to be the key phenolic compounds to differentiate ‘Duoju’ from ‘Taiju’.

## 1. Introduction

*Chrysanthemum morifolium* Ramat belongs to the family *Asteraceae*, and most of these flowering plants are widely planted in East Asia and northeastern Europe [1,2]. It is called ‘Ju Hua’ in Chinese, and has been used as herbal medicine in many Asian countries, including China, Japan, South Korea and Thailand [3,4]. It is reported in Chinese Pharmacopoeia that drinking *C. morifolium* tea (infusion) could help alleviating headache and preventing the occurrence of cold and fever [5,6,7,8]. Additionally, drinking *C. morifolium* tea has the potential to improve the eye function [5,6,7,9]. 

Recent pharmacological studies demonstrate that *C. morifolium* possesses a wide array of health potentials, such as anti-bacterial, anti-viral and anti-inflammatory capacities [4,6,8,9,10,11,12]. During its long evolution, ‘Hangju’, ‘Boju’, ‘Chuju’, and ‘Gongju’ are the four main varieties of *C. morifolium* cited in the Chinese Pharmacopeia [7]. *C. morifolium* has been reported to contain numerous bioactive compounds such as phenolics, which possess antioxidant activity [1,13]. Up to date, the main phenolic compounds in *C. morifolium* include 5-*O*-caffeoylquinic acid (chlorogenic acid), 3-*O*-caffeoylquinic acid, 4-*O*-caffeoylquinic acid, 3,4-di-*O*-caffeoylquinic acid, 3,5-di-*O*-caffeoylquinic acid, 4,5-di-*O*-caffeoylquinic acid, caffeic acid, luteolin, luteolin-7-*O*-glucopyranoside, apigenin, apigenin-7-*O*-glucoside, acacetin-7-*O*-rutinoside, acacetin [3,4,5,11,12,13,14,15,16]. Among these compounds, 5-*O*-caffeoylquinic acid and 3,5-di-*O*-caffeoylquinic acid are representative caffeoylquinic acids that significantly affect the antioxidant activity of *C. morifolium* [1,3]. According to Chinese Pharmacopoeia, the content of 5-*O*-caffeoylquinic acid, 3,5-di-*O*-caffeoylquinic acid and luteoloside (luteolin-7-*O*-glucoside) should be above 0.2 g/100 g DW, 0.7 g/100 g DW and 0.08 g/100 g DW, respectively to qualify *C. morifolium* as a herbal medicine [7].

‘Hangbaiju’ is a cultivar of *Chrysanthemum morifolium* Ramat, which is mainly planted in Zhejiang Province, and has been widely used to prepare a functional beverage in China due to its unique sensory attributes and multiple health beneficial features [6]. ‘Hangbaiju’ can be further classified into two mainly commercial products, including ‘Duoju’ (named ‘DJ’) and ‘Taiju’ (named ‘TJ’), according to their different harvest time [13]. ‘Duoju’ represents the dried flower heads harvested with their ray florets and tubular florets being fully opened (harvested in November), whereas ‘Taiju’ are the dried flower heads with opened ray florets but closed tubular florets (harvested in October) [13]. We hypothesized that ‘Duoju’ and ‘Taiju’ may possess different phenolic compositions due to their different harvest seasons, which could affect their antioxidant properties. To this end, 6 ‘Duoju’ and 9 ‘Taiju’ were selected for the present study. Their phenolic compositions and antioxidant properties were analyzed and compared. Additionally, multivariate statistical analysis (principal component analysis) was applied to differentiate these ‘Hangbaiju’ based on their phenolic profiles. This study could provide useful information on the quality control of ‘Hangbaiju’ in China.

## 2. Materials and Methods

### 2.1. Materials

A total of 6 ‘Duoju’ and 9 ‘Taiju’ were collected in multiple supermarkets in Hangzhou, Zhejiang, China. ‘DJ1′, ‘DJ2′, ‘DJ3′ and ‘DJ4′ samples were planted in Tongxiang, whereas the ‘DJ5′ sample was cultivated in Jiaxing and our ‘DJ6′ sample in Hangzhou, Zhejiang, China. Regarding the ‘Taiju’ samples, ‘TJ1′, ‘TJ2′, ‘TJ3′ and ‘TJ4′ were from the Tongxiang area, whereas these ‘TJ5′ to ‘TJ8′ samples were from the Jiaxing area of Zhejiang. The ‘TJ9′ sample was collected from Bozhou, Anhui Province, China.

### 2.2. Chemicals

Chemical standards, including apigenin-7-*O*-glucoside, 3-*O*-caffeoylquinic acid, 4-*O*-caffeoyl-quinic acid, 3,5-di-*O*-caffeoylquinic acid, 4,5-di-*O*-caffeoylquinic acid, 3,4-di-*O*-caffeoylquinic acid, luteoloside and linarin were purchased from Shanghai Yongheng Biotechnology Co. Ltd. (Shanghai, China) with a purity of at least 98%. The standards of hyperoside and acacetin, with a purity of 98.2% and 98.5 % respectively, were from Shanghai Ronghe Medical Biotechnology Co. Ltd. (Shanghai, China). The standards of 5-*O*-caffeoylquinic acid (98.8%) and caffeic acid (98.6%), 6-Hydroxy-2,5,7,8-tetramethylchroman-2-carboxylic acid (Trolox) and Vitamin C were purchased from J&K Scientific Ltd. (Shanghai, China). Apigenin (98.5%) and luteolin (98.3%) standards were received from Zhejiang Tiancao Biotechnology Co. Ltd. (Zhejiang, China). 1,1-Diphenyl-2-picrylhydrazyl (DPPH), 2,2′-Azinobis(3-ethylbenzothiazoline-6-sulphonic acid) diammonium salt (ABTS) and 2,4,6-Tri(2-pyridyl)-1,3,5-triazine (TPTZ) were purchased from Tokyo Chemical Industry Development Co. Ltd. (Shanghai, China). 

Phosphoric acid was of analytical grade and purchased from Sinopharm Chemical Reagent Co. Ltd. (Shanghai, China), whereas the HPLC grade acetonitrile and methanol were purchased from Merck (Darmstadt, Germany). Water was obtained from a MilliQ purification system (Bedford, MA, USA).

### 2.3. Sample Extraction

The ‘Hangbaiju’ samples were ground into powder by a Chinese medicine crusher (Shanghai Dianjiu Traditional Chinese Medicine Machinery Manufacturing Co. Ltd., Shanghai, China) and passed through a 60 mesh sieve. About 0.5 g sample weighed with the precision of 0.1 mg using an AB-135s scale (METTLER TOLEDO), which was mixed with 100 mL of methanol:water (70:30 *v*/*v*) in a glass tube, and sonicated at 50 °C for 40 min. Afterwards, the mixture was cooled to room temperature, and the final extract volume was brought up to 100 mL using the methanol:water solution [7]. The extract was filtered through a 0.22 µm PTFE membrane and stored at −20 °C for further analysis.

### 2.4. HPLC-DAD Analysis

A Waters E2695 HPLC coupled with a 2998 diode array detector (Waters, Milford, MA, USA) was used to analyze the phenolic compounds in the ‘Hangbaiju’ samples. A Phenomenex Luna C_18_ column (250 mm × 4.6 mm, 5 µm, Torrance, CA, USA) was used to separate the phenolic compounds under a 0.8 mL/min flow rate. The mobile phase consisted of (A) acetonitrile and (B) 0.1% phosphoric acid in water (*v*/*v*). The sample injection volume was 10 µL, and the column was maintained at 35 °C during the elution program. The gradient was programed as follows, 0 to 11 min, 10% A to 18% A; 11 to 32 min, 18% A isocratic; 32 to 40 min, 18% A to 30% A; 40 to 48 min, 30% A to 35% A; 48 to 50 min, 35% A to 40% A; 50 to 55 min, 40% A isocratic; 55 to 60 min, 40% A to 70% A; and 60 to 70 min, 70% A to 10% A. Phenolic compounds were identified by comparing their retention time with corresponding external standards. The quantification was also conducted using the standards.

### 2.5. DPPH Assay

The antioxidant activity of the ‘Hangbaiju’ extracts and standard solutions using the DPPH assay followed the method of Turkoglu et al. [17] with minor modifications. Briefly, 20 mg of DPPH was dissolved in methanol in a 500 mL volumetric flask to a concentration of 0.101 M. The extract or standard solution (0.2 mL) was mixed with 3.8 mL of the DPPH solution. The mixture was well vortexed and kept at room temperature for 1 h. Afterwards, the absorbance of the mixture was recorded at 517 nm on a UV-5200PC spectrophotometer (Metash instrument, Shanghai, China). The reference was prepared by mixing 0.2 mL of 50% methanol solution with 3.8 mL DPPH solution. The DPPH radical inhibition rate of these samples was calculated using the equation below,

Inhibition rate = (A_ref_ − A_sample_)/A_ref_ × 100% (1)

where A_ref_ and A_sample_ were the absorbance of the reference and the sample, respectively. EC_50_ represents the sample with its concentration that inhibits 50% of DPPH radicals. Each measurement was carried out in triplicate.

### 2.6. ABTS Assay

The ABTS assay was followed the method of Re et al. [18] with minor modifications. In brief, 176 µL of 140 mM potassium persulfate solution was mixed with 10 mL of 7 mM ABTS solution to yield ABTS working solution. The working solution was kept in the dark for 12 h. During the analysis, the ABTS working solution was diluted by ethanol to an absorbance value of 0.70 ± 0.02 at 734 nm on the UV-5200PC spectrophotometer. Afterwards, 0.1 mL extract or standard solution was mixed with 3.9 mL ABTS working solution and kept at room temperature for 6 min in the dark. The absorbance of the mixture was also recorded at 734 nm. 

The 50% methanol solution (0.1 mL) mixed with 3.9 mL ABTS working solution was used as the reference. The ABTS radical inhibition rate of the sample was calculated using the equation below,

Inhibition rate = (A_ref_ − A_sample_)/A_ref_ × 100% (2)

where A_ref_ and A_sample_ were the absorbance of the reference and the sample, respectively. EC_50_ was used to represent the inhibition activity of each sample against 50% of the ABTS radical. Each measurement was conducted in triplicate.

### 2.7. Ferric Reducing Antioxidant Power (FRAP) Assay

FRAP assay was carried out according to Benzie & Strain [19] with minor modifications. In brief, the FRAP working solution was prepared by mixing 0.1 M acetate solution (pH 3.6, acidic), 10 mM TPTZ solution and 20 mM ferric chloride solution at a 10:1:1 (*v*/*v*/*v*) ratio. Afterwards, 0.1 mL extract sample or standard solution was mixed with 3.9 mL FRAP working solution and vortexed. The resultant mixture was incubated at a 37 °C water bath for 10 min. After cooling to room temperature, the absorbance of the mixture was recorded at 593 nm. The reference was prepared by mixing 50% methanol with 3.9 mL FRAP working solution. Trolox was used as the external standard. The FRAP value of each sample was expressed as mg TEAC/g dry weight. Each analysis was performed in triplicate.

### 2.8. Statistical Analysis

All data were expressed as the mean ± standard deviation of triplicate tests. Analysis of variance (ANOVA) was carried out to investigate the significant difference among the means at a significant level of 0.05 using SPSS22.0 software (SPSS Inc, Chicago, IL, USA). Principal component analysis (PCA) (Metabo Analyst 4.0, http://www.metaboanalyst.ca) was carried out using phenolic compounds as variable to elucidate the similarity of ‘Hangbaiju’ samples.

## 3. Results

### 3.1. Phenolic Composition

**Figure 1:**
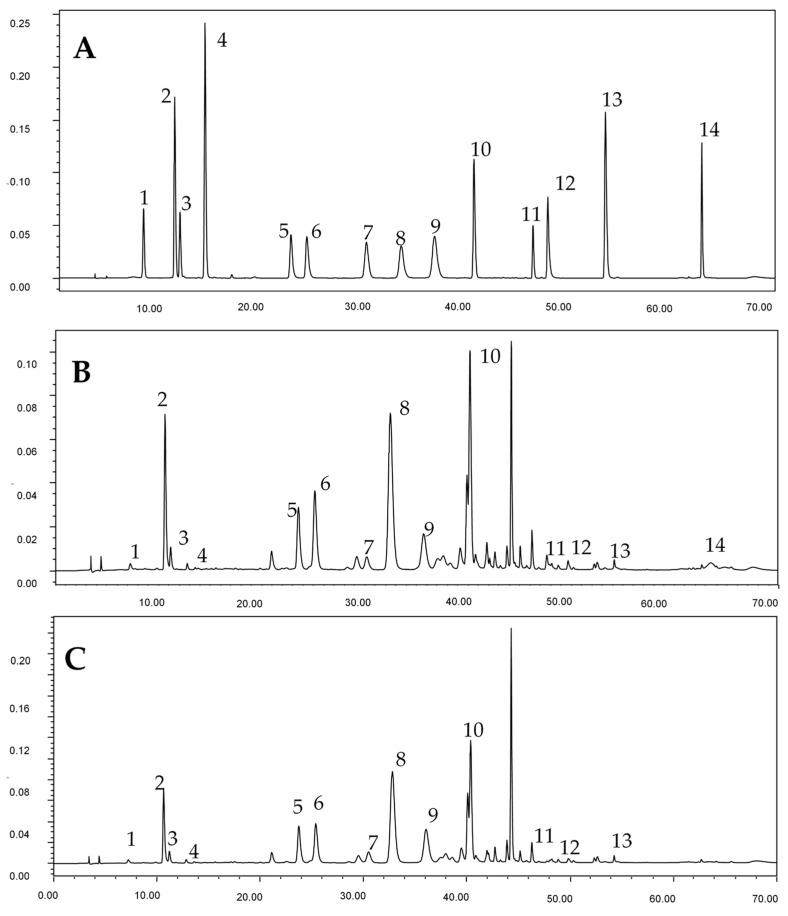
Chromatography of phenolic compounds of (**A**) standards, (**B**) ‘DJ2′ sample and (**C**) ‘TJ4′ sample. Peak 1, 2, 3, 4, 5, 6, 7, 8, 9, 10, 11, 12, 13 and 14 represent 3-*O*-caffeoylquinic acid, 5-*O*-caffeoylquinic acid, 4-*O*-caffeoylquinic acid, caffeic acid, hyperoside, luteoloside, 3,4-di-*O*-caffeoylquinic acid, 3,5-di-*O*-caffeoylquinic acid, apigenin-7-*O*-glucoside, 4,5-di-*O*-caffeoylquinic acid, linarin, luteolin, apigenin and acacetin, respectively.

Figure 1 shows the chromatography of phenolic compounds of standards and representative “Hangbaiju” samples of ‘DJ2′ and ‘TJ4′. Phenolic acids and flavonoids are two groups of active substances in ‘Hangbaiju’. The contents of individual phenolic compounds and the total mono-caffeoylquinic acid contents (TMAC, the sum of 3-*O*-caffeoylquinic acid, 5-*O*-caffeoylquinic acid, and 4-*O*-caffeoylquinic acid), along with the total di-caffeoylquinic acid contents (TDAC, the sum of 3,5-di-*O*-caffeoylquinic acid, 4,5-di-O-caffeoylquinic acid and 3,4-di-*O*-caffeoylquinic acid), the total phenolic acid contents (TPAC, the sum of 3-*O*-caffeoylquinic acid, 5-*O*-caffeoylquinic acid, 4-*O*-caffeoylquinic acid, caffeic acid, 3,4-di-*O*-caffeoylquinic acid, 4,5-di-O-caffeoylquinic acid, 3,5-di-*O*-caffeoylquinic acid) as well as the total flavonoids contents (TFC, the sum of hyperoside, luteoloside, apigenin-7-*O*-glucoside, linarin, luteolin, apigenin and acacetin) and three key phenolic components (TKPC, the sum of 5-*O*-caffeoylquinic acid, 3,5-di-*O*-caffeoylquinic acid and luteoloside), in these samples, are listed in Table 1 and Table 2. 

The TMAC was found to be in the range of 2552.04 to 7402.83 µg/g DW in 15 ‘Hangbaiju’ samples, in which the ‘Duoju’ samples had the content between 2552.04 and 4352.29 µg/g DW and the ‘Taiju’ samples between 2673.11 and 7402.83 µg/g DW. In comparison of the TMAC among ‘Duoju’, ‘DJ6′ was higher than others (*P* < 0.05). The contents of 3-*O*-caffeoylquinic acid, 5-*O*-caffeoylquinic acid and 4-*O*-caffeoylquinic acid in the ‘DJ6′ were found to be 1167.59 µg/g DW, 2392.17 µg/g DW and 792.53 µg/g DW, respectively, which were also significantly higher than the other ‘Duoju’ (*P* < 0.05). It is worth noting that 5-*O*-caffeoylquinic acid was the dominant mono-caffeoylquinic acid in the ‘Duoju’ samples. These results were consistent with a previous report [13]. Among the ‘Taiju’ samples, ‘TJ3′ had the highest TMAC (*P* < 0.05), with 2844.64 µg/g DW of 3-*O*-caffeoylquinic acid, 3139.04 µg/g DW of 5-*O*-caffeoylquinic acid and 1365.16 µg/g DW of 4-*O*-caffeoylquinic acid, respectively. 5-*O*-Caffeoylquinic acid was also the predominant individual mono-caffeoylquinic acid in the ‘Taiju’ samples. Different to ‘Duoju’, the 3-*O*-caffeoylquinic acid content in ‘Taiju’ was much higher than 4-*O*-caffeoylquinic acid (Table 1 and Table 2).

The TDAC in the 6 ‘Duoju’ samples were between 8153.62 µg/g DW and 10,974.94 µg/g DW, and in the 9 ‘Taiju’ samples between 7718.79 to 13,960.39 µg/g DW (Table 1 and Table 2). It was observed that 7 out of 9 ‘Taiju’ samples had higher TDAC than the highest content ‘Duoju’ (‘DJ6′, 10,974.94 µg/g DW) sample. Among the 6 ‘Duoju’ samples, the content of 3,4-di-*O*-caffeoylquinic acid in the ‘DJ6′ were significantly higher than the rest, whereas the content of 3,5-di-*O*-caffeoylquinic acid in the ‘DJ2′ and ‘DJ6′, and content of 4,5-di-*O*-caffeoylquinic acid in the ‘DJ5′and ‘DJ6′ were significantly higher than the others (Table 1). In both ‘Duoju’ and ‘Taiju’ samples, 3,5-di-*O*-caffeoylquinic acid was the dominant individual di-caffeoylquinic acid, followed by 4,5-di-*O*-caffeoylquinic acid and then 3,4-di-*O*-caffeoylquinic acid. ‘TJ5′ contained the highest 3,5-di-*O*-caffeoylquinic acid and 4,5-di-*O*-caffeoylquinic acid among the tested 15 ‘Hangbaiju’ samples. Regarding caffeic acid content in the ‘Taiju’ samples, ‘TJ3′ possessed the highest content (23.03 µg/g DW), but it was not detected in ‘TJ1′ (Table 2). Among the ‘Duoju’ samples, the highest caffeic acid content was found in the ‘DJ1′ (10.85 µg/g DW), but it was not detected in ‘DJ4′ and ‘DJ6′ (Table 1). 

The TPAC in ‘Taiju’ was ranged between 10,405.31 µg/g DW and 20,847.76 µg/g DW, and in ‘Duoju’ between 10,709.58 µg/g DW and 15,327.22 µg/g DW, respectively (Table 1 and Table 2). It should be noted that the highest and lowest TPAC was all found in ‘Taiju’ samples, and 7 out of 9 ‘Taiju’ samples had higher TPAC than the highest TPAC ‘Duoju’ (DJ6, 15,327.22 µg/g DW) sample. Among the 6 ‘Duoju’ samples, ‘DJ2′ showed significantly higher TPAC than ‘DJ3′ and ‘DJ4′ (*P* < 0.05), but no significant differences were observed between ‘DJ2′ with the rest of the ‘Duoju’ samples. Among the ‘Taiju’ samples, ‘TJ3′ had significantly higher TPAC than all of the others (*P* < 0.05).

In terms of flavonoids, these ‘Hangbaiju’ samples contained hyperoside, luteoloside, apigenin-7-*O*-glucoside, linarin, luteolin, apigenin and acacetin (Table 1 and Table 2). The TFC was ranged from 5183.35 to 9792.01 µg/g DW, and ‘Taiju’ had higher TFC than that of ‘Duoju’. Regarding the individual flavonoids, all samples contained hyperoside, luteoloside, apigenin-7-O-glucoside, linarin and luteolin. Specifically, ‘DJ5′ had the highest content of hyperoside (813.68 µg/g DW) and linarin (2605.87 µg/g DW). Both ‘DJ5′ and ‘DJ2′ showed similar content on luteolin (247.09 µg/g DW and 238.37 µg/g DW, respectively), and ‘DJ3′ and ‘DJ4′ contained similar amount of luteoloside (1524.16 µg/g DW and 1476.67 µg/g DW, respectively). ‘DJ4′ showed the highest content of apigenin-7-*O*-glucoside among the ‘Duoju’ (*P* < 0.05). The contents of luteoloside and linarin in all most ‘Taiju’ were higher than most ‘Duoju’ samples, whereas the contents of luteolin in all ‘Taiju’ were less than all ‘Duoju’ samples (Table 1 and Table 2). ‘TJ3′ showed the highest hyperoside content (994.81 µg/g DW), and also showed significant higher luteolin content than other ‘Taiju’ (*P* < 0.05). ‘TJ9′ contained the highest content of luteoloside and apigenin-7-*O*-glucoside (2416.07 µg/g DW and 3539.55 µg/g DW), whereas the highest content of linarin was found in ‘TJ6′ (3573.51 µg/g DW) and ‘TJ3′ (3470.66 µg/g DW). It should be noted that apigenin was only present in 3 out of 6 ‘Duoju’ samples and 7 out of 9 ‘Taiju’ samples. All ‘Taiju’ did not contain acacetin, which was only found in ‘Duoju’, and ‘DJ6′ possessed the highest content (29.48 µg/g DW). 

It has been reported that 5-*O*-caffeoylquinic acid, 3,5-di-*O*-caffeoylquinic acid and luteoloside were the most crucial phenolic components in the chrysanthemum that influence the antioxidant and medicinal properties [7,13]. Therefore, it is important to compare the total content of these three key phenolic components. Among these 15 ‘Hangbaiju’ samples, the TKPC was ranged from 7591.50 µg/g DW to 13,144.12 µg/g DW. In the ‘Duoju’ samples, ‘DJ6′ and ‘DJ2′ contained the highest TKPC. It was observed that 7 ‘Taiju’ samples (TJ1, TJ3, TJ4, TJ5, TJ6, TJ8 and TJ9) had higher TKPC than any of the ‘Duoju’ samples. It should be noticed that ‘TJ3′ and ‘TJ5′ had similar TKPC values and were significantly higher than the other ‘Taiju’ samples (*P* < 0.05).

### 3.2. Antioxidant Activity

DPPH, ABTS and FRAP assays are often used simultaneously to evaluate the in vitro antioxidant activities of extracts or active substances [20,21,22]. Table 3 and Table 4 show the antioxidant activity of these ‘Hangbaiju’ samples. In the DPPH assay, antioxidants work as hydrogen donors to react with stable DPPH free radical causing its discoloration [23,24]. The EC_50_ of DPPH of these samples ranged from 1.69 mg/L to 3.04 mg/L. ‘DJ2′ exhibited the highest DPPH scavenging activity among the ‘Duoju’ (*P* < 0.05) samples, whereas ‘TJ6′, ‘TJ4′, ‘TJ1′ and ‘TJ8′ had the strongest DPPH scavenging capacity among ‘Taiju’ samples. In the ABTS assay, the stable-colored ABTS radicals are interact with antioxidants and this results in color loss [14,24]. In the present study, the ‘Duoju’ samples had the ABTS quenching EC_50_ value of 2.13 mg/mL to 2.83 mg/mL, and between 1.82 mg/mL to 2.42 mg/mL for ‘Taiju samples’. The highest ABTS quenching activity of ‘Taiju’ samples was found in ‘TJ4′, whereas ‘TJ1′, ‘TJ5′, ‘TJ6′ and ‘TJ8′ possessed similar ABTS radicals scavenging capacity. FRAP assay is used to estimate the antioxidant activity of a compound through its capacity of reducing ferric into ferrous ions [14]. In this study, the ‘Hangbaiju’ samples had the FRAP value of 222.28 to 436.51 mg TEAC/g DW, and generally the ‘Taiju’ samples exhibited a higher reduction capacity than did the ‘Duoju’ samples. Among these ‘Duoju’ samples, ‘DJ1′ and ‘DJ6′ had the lowest and highest FRAP reduction capacity, respectively. The highest FRAP reduction ability among ‘Taiju’ was ‘TJ8′ (436.51 mg TEAC/g DW), and the ‘TJ9′ showed the lowest FRAP value.

It has been reported that more hydroxyl groups in the flavonoid molecular structure could enhance the antioxidant capacity [25]. Additionally, the acylation of caffeoyl group could improve the complexity of the caffeoylquinic acid, resulting in a stronger capacity of scavenging free radicals [26]. The antioxidant activities of individual phenolic components from these ‘Hangbaiju’ samples were also evaluated (Table 5). Hyperoside, luteolin, 4,5-di-*O*-caffeoylquinic acid, luteoloside, 3,4-di-*O*-caffeoylquinic acid, 3,5-di-*O*-caffeoylquinic acid, caffeic acid and 5-*O*-caffeoylquinic acid show similar DPPH radical scavenge capacity, but they are higher than that of 4-*O*-caffeoylquinic acid, 3-*O*-caffeoylquinic acid, apigenin, apigenin-7-*O*-glucoside and acacetin. However, the DPPH radical scavenging capacity of linarin was not detected.

In terms of ABTS radical scavenging capacity, 4,5-di-*O*-caffeoylquinic acid, 3,4-di-*O*-caffeoylquinic acid, 3,5-di-*O*-caffeoylquinic acid, luteolin, 5-*O*-caffeoylquinic acid, caffeic acid, 4-*O*-caffeoylquinic acid, hyperoside and luteoloside show similar values, but their ABTS radical scavenging activity was greater than that of 3-*O*-caffeoylquinic acid, apigenin, apigenin-7-*O*-glucoside, acacetin and linarin. The strongest FRAP reduction activity was found to be 5-*O*-caffeoylqunic acid, which was much higher than other phenolic compounds. Additionally, 3,4-di-*O*-caffeoylquinic acid and 4,5-di-*O*-caffeoylquinic acid also show great FRAP reduction ability, followed by 4-*O*-caffeoylquinic acid, 3-*O*-caffeoylquinic acid, 3,5-di-*O*-caffeoylquinic acid and caffeic acid. Flavonoids used in the present study show relatively weak FRAP reduction activity, which is in the order of hyperoside > luteolin > luteoloside > apigenin > apigenin-7-*O*-glucoside > acacetin > linarin.

It has been reported that phenolic compounds play a vital role to the antioxidant activity in *C. morifolium* [3]. A correlation study was conducted between the phenolic compounds and the antioxidant capacities of these ‘Hangbaiju’ samples (Table 6). It was observed that phenolic compounds in both ‘Duoju’ and ‘Taiju’ exhibit a good correlation with their antioxidant activities. For example, a positive correlation was established between 5-*O*-caffeoylquinic acid, TPAC and TKPC with the DPPH and ABTS quenching activity in the ‘Duoju’. However, such a correlation was weak for ‘Taiju’. In the FRAP assay system, TMAC and 5-*O*-caffeoylquinic acid showed a weak correlation with the FRAP reducing capacity. This indicates that phenolic acids including 5-*O*-caffeoylquinic acid, 3,5-di-*O*-caffeoylquinic acid and luteoloside might be the key compounds that contribute to the antioxidant activity of these ‘Hangbaiju’ samples.

### 3.3. Principal Component Analysis (PCA)

**Figure 2:**
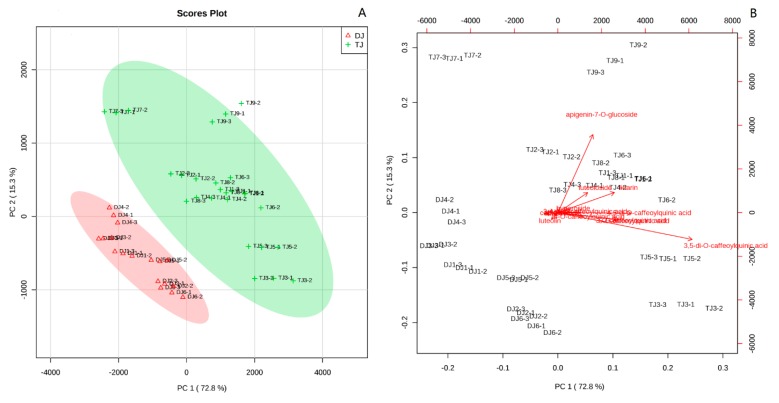
(**A**) Score plot of principal component analysis. DJ and TJ represent ‘Duoju’ and ‘Taiju’ samples. (**B**) Loading plot of principal component analysis. DJn-1, DJn-2 and DJn-3 represent triplicate independent experiments of DJn (n range is 1-6). TJm-1, TJm-2 and TJm-3 represent triplicate independent experiments of TJm (m range is 1-9).

PCA is commonly used to explain differentiation between samples and to obtain information on the variables that mainly influence the sample similarities and differences [27]. In order to differentiate these ‘Hangbaiju’, PCA was carried out using the detected phenolic compounds as variables. The first component (PC1) and second component (PC2) accounted for 72.8% and 15.3% of the total variance, respectively (Figure 2A). The ‘Duoju’ were aggregated together on the left downside of the score plot and segregated from the ‘Taiju’. According to the loading plot (Figure 2B), apigenin-7-*O*-glucoside and 3,5-di-*O*-caffeoylquinic acid had significant differences between the ‘Duoju’ and the ‘Taiju’ samples. Meanwhile, the content of luteolin in the ‘Duoju’ was about 3–4 times higher than that in the ‘Taiju’. Therefore, luteolin also played a vital role in segregating ‘Taiju’ and ‘Duoju’ (Table 1 and Table 2). It should be noted that acacetin was only present in some ‘Duoju’, but ‘Taiju’ contained a higher content of apigenin-7-*O*-glucoside than that of ‘Duoju’. Therefore, acacetin and luteolin also affected the differentiation between ‘Taiju’ and ‘Duoju’ (Table 1 and Table 2).

## 4. Discussion

Phenolic acids and flavonoids are two groups of active substances in ‘Hangbaiju’, especially the caffeoylquinic acid. Although 5-*O*-caffeoylquinic acid and 3,5-di-*O*-caffeoylquinic acid play an absolute dominant role in mono-caffeoylquinic acids and di-caffeoylquinic acids, respectively. Mono-caffeoylquinic acids can be converted to each other, as well as di-caffeoylquinic acid [28,29,30], therefore total mono-caffeoylquinic acid contents (TMAC) and total di-caffeoylquinic acid contents (TDAC) were used, rather than the content of single mono-caffeoylquinic acid or di-caffeoylquinic acid.

The flavonoid contents in *C. morifolium* rose during the flowering period and then declined; the general trend was a “peak shape” degree of flower openness [3]. The “peak shape” course may relate to the key enzyme in flavonoid biosynthesis—chalcone isomerase (CHI). During early florescence, CHI activity was enhanced, and the flavonoids gradually accumulated; at the end of florescence, CHI activity was inhibited, and the flavonoids gradually decreased [6,31]. ‘Taiju’ are the dried flower heads with opened ray florets but closed tubular florets, and CHI activity was enhanced, and the flavonoids gradually accumulated. But ‘Duoju’ represent the dried flower heads harvested with their ray florets and tubular florets being fully opened, so CHI activity was inhibited, and the flavonoids gradually decreased. Most contents of flavonoids in ‘Taiju’ are higher than in ‘Duoju’.

It has been required by the Chinese Pharmacopoeia that the content of 5-*O*-caffeoylquinic acid, 3,5-di-*O*-caffeoylquinic acid and luteoloside in *C. morifolium* should be above 0.2 g/100 g DW, 0.7 g/100 g DW and 0.08 g/100 g DW to exhibit strong antioxidant activity and medicinal property [7]. In the present study, three ‘Duoju’ samples, namely ‘DJ2′, ‘DJ5′ and ‘DJ6′, had more than 0.2 g/100 g DW of 5-*O*-caffeoylquinic acid and more than 0.08 g/100 g DW of luteoloside. However, these ‘Duoju’ contained less than 0.7 g/100 g DW of 3,5-di-*O*-caffeoylquinic acid content (Table 1). Therefore, these ‘Duoju’ were not qualified as a Chinese herb medicine according to the Chinese Pharmacopoeia. However, ‘TJ1′, ‘TJ3′, ‘TJ4′, ‘TJ5′, ‘TJ6′ and ‘TJ8′ can be claimed as Chinese herb medicines, because they have met the above criteria (Table 2). It was reported that different drying methods affected the phytochemicals contents, including 5-*O*-caffeoylquinic acid, luteolin-7-*O*-glucoside, 3,5-di-caffeoylquinic acid, apigenin-7-*O*-glucopyranoside, luteolin, acacetin-7-*O*-glucopyranoside, apigenin, acacetin and the antioxidant properties of chrysanthemum flower heads. In this study, 5-*O*-caffeoylquinic acid and 3,5-di-caffeoylquinic acid contents in some “Hangbaiju” samples were lower than the threshold of 0.2 g/100 g DW and 0.7 g/g DW of Chinese Pharmacopoeia, respectively. However, the level of luteolin-7-*O*-glucoside in all tested samples was significantly higher than the level of 0.08 g/100 g DW [13], which is consistent with our results.

Linarin (acacetin-7-*O*-rutinoside), luteoloside (luteolin-7-*O*-glucoside) and apigenin-7-*O*-glucoside are the glycoside of acacetin, luteolin and apigenin, respectively. Compared with apigenin, luteolin has a hydroxyl at the 3′ position, and compared with linarin, the 4′ hydroxyl of apigenin was methylated. Bobilya suggested that glycosidation and methylation of the hydroxyl group results in a decreased antioxidant activity of flavonoids [32]. Numerous studies have shown that the increase of hydroxyl groups in flavonoids can increase their antioxidant activity, especially the formation of o-dihydroxyl groups at the 3′ and 4′ positions [32,33,34]. Our study also echoes this claim. In the three antioxidant systems, luteolin showed higher antioxidant activity than apigenin, followed by acacetin, and luteoloside had higher antioxidant activity than apigenin-7-*O*-glucoside and linarin.

## 5. Conclusions

In conclusion, phenolic compounds and antioxidant capacity were analyzed and compared in 15 ‘Hangbaiju’, including 6 ‘Duoju’ and 9 ‘Taiju’. The compositions of phenolic compounds were significantly different between ‘Duoju’ and ‘Taiju’. ‘Taiju’ contained a higher content of caffeoylquinic acids and higher antioxidant activities than ‘Duoju’, suggesting that the flowers harvested earlier had higher phenolic contents and antioxidant activity. A correlation study indicated that phenolic compounds, especially caffeoylquinic acids, played a vital role to the antioxidant capacity. Principal component analysis indicates that the difference on the phenolic composition could be used to differentiate ‘Duoju’ from ‘Taiju’ of *C. morifolium*.

## Figures and Tables

**Table 1:** Contents of phenolic compounds in different ‘Duoju’ (μg/g DW).

Phenolic Compound	DJ1	DJ2	DJ3	DJ4	DJ5	DJ6
3-*O*-caffeoylquinic acid	691.48 ± 21.98 ^d^	889.07 ± 25.98 ^c^	597.64 ± 19.87 ^e^	644.24 ± 30.34 ^e^	965.49 ± 38.17 ^b^	1167.59 ± 16.22 ^a^
5-*O*-caffeoylquinic acid	1745.29 ± 55.86 ^c^	2083.55 ± 81.66 ^b^	1413.66 ± 65.66 ^d^	1370.96 ± 54.98 ^d^	2189.24 ± 58.96 ^b^	2392.17 ± 136.86 ^a^
4-*O*-caffeoylquinic acid	640.83 ± 30.17 ^b^	765.78 ± 36.74 ^a^	540.74 ± 15.36 ^c^	521.76 ± 19.27 ^c^	764.92 ± 20.67 ^a^	792.53 ± 13.98 ^a^
TMAC	3077.60 ± 70.87 ^c^	3738.40 ± 108.99 ^b^	2552.04 ± 70.11 ^d^	2536.96 ± 58.78 ^d^	3919.65 ± 69.86 ^b^	4352.29 ± 140.11 ^a^
3,4-di-*O*-caffeoylquinic acid	282.62 ± 9.94 ^c^	379.66 ± 15.67 ^b^	259.12 ± 6.98 ^d^	289.24 ± 18.27 ^c^	385.30 ± 17.18 ^b^	530.91 ± 9.85 ^a^
3,5-di-*O*-caffeoylquinic acid	5275.20 ± 210.76 ^c^	6460.38 ± 201.38 ^a^	4736.66 ± 286.99 ^d^	4918.72 ± 229.38 ^cd^	5796.97 ± 198.35 ^b^	6421.88 ± 298.67 ^a^
4,5-di-*O*-caffeoylquinic acid	3156.97 ± 96.38 ^c^	3702.25 ± 109.26 ^b^	3158.17 ± 157.88 ^c^	3087.61 ± 69.20 ^c^	3880.21 ± 158.29 ^ab^	4022.14 ± 156.76 ^a^
TDAC	8714.80 ± 250.49 ^a^	10542.71 ± 240.56 ^a^	8153.62 ± 300.37 ^a^	8295.57 ± 248.27 ^a^	10062.48 ± 210.85 ^a^	10974.94 ± 371.48 ^a^
caffeic acid	10.85±0.36^a^	9.11 ± 0.33 ^b^	3.92 ± 0.55 ^c^	-	10.71 ± 0.32 ^a^	-
TPAC	11803.25 ± 281.19 ^ab^	14290.22 ± 281.01 ^a^	10709.58 ± 328.27 ^b^	10832.53 ± 260.99 ^b^	13992.84 ± 241.33 ^ab^	15327.22 ± 430.98 ^ab^
hyperoside	655.32 ± 25.11 ^c^	739.11 ± 30.64 ^b^	613.70 ± 40.48 ^c^	628.19 ± 12.36 ^c^	813.68 ± 20.66 ^a^	666.99 ± 10.38 ^c^
luteoloside	1135.19 ± 40.14 ^d^	1335.69 ± 42.11 ^c^	1524.16 ± 65.83 ^a^	1476.67 ± 45.21 ^ab^	1407.85 ± 50.74 ^bc^	1369.87 ± 20.18 ^c^
apigenin-7-*O*-glucoside	1293.56 ± 30.85 ^bc^	1264.53 ± 49.76 ^bc^	1302.82 ± 89.27 ^b^	1593.54 ± 40.16 ^a^	1211.42 ± 30.34 ^c^	1234.12 ± 31.73 ^c^
linarin	2057.88 ± 87.21 ^c^	1955.80 ± 59.75 ^c^	1647.05 ± 61.85 ^d^	2194.81 ± 39.11 ^b^	2605.87 ± 87.83 ^a^	1675.81 ± 20.11 ^d^
luteolin	200.10 ± 8.86 ^cd^	238.37 ± 9.28 ^a^	191.73 ± 3.59 ^d^	219.25 ± 4.19 ^b^	247.09 ± 15.22 ^a^	207.07 ± 4.76 ^bc^
apigenin	63.36 ± 1.87 ^b^	-	-	36.44 ± 0.54 ^c^	73.87 ± 2.27 ^a^	-
acacetin	4.67 ± 0.13 ^c^	-	13.87 ± 0.40 ^b^	3.9 ± 0.10 ^c^	15.07 ± 0.41 ^b^	29.48 ± 0.67 ^a^
TFC	5410.06 ± 98.37 ^bc^	5533.50 ± 25.87 ^b^	5293.33 ± 78.28b ^c^	6152.90 ± 50.38 ^a^	6374.85 ± 63.19 ^a^	5183.35 ± 50.67 ^c^
TKPC	8155.68 ± 321.12 ^c^	9880.04 ± 438.21 ^ab^	7674.15 ± 381.37 ^c^	7766.35 ± 391.5 ^c^	9394.06 ± 411.82 ^b^	10183.92 ± 501.02 ^a^

Data are the mean ± standard deviation of triplicate independent experiments. “-” represents ‘not detected’. ^a, b, c, d, e^ Different letters in each raw indicate significant difference at a significant level of 0.05. TMAC: Total mono-caffeoylquinic acid contents, TDAC: Total di-caffeoylquinic acid contents, TPAC: Total phenolic acid contents, TFC: Total flavonoids contents, TKPC: Three key phenolic components contents.

**Table 2:** Contents of phenolic compounds in different ‘Taiju’ (μg/g DW).

Phenolic Compound	TJ1	TJ2	TJ3	TJ4	TJ5	TJ6	TJ7	TJ8	TJ9
3-*O*-caffeoylquinic acid	1555.13 ± 50.01 ^c^	1246.00 ± 40.11 ^e^	2844.64 ± 66.38 ^a^	1117.50 ± 37.88 ^f^	1641.14 ± 47.32 ^b^	1589.85 ± 59.38 ^bc^	673.09 ± 28.93 ^g^	1403.69 ± 38.29 ^d^	1560.38 ± 50.21 ^c^
5-*O*-caffeoylquinic acid	2649.68 ± 60.19 ^cd^	2180.97 ± 182.93 ^e^	3193.04 ± 167.95 ^a^	2205.75 ± 129.84 ^e^	2759.82 ± 164.23 ^bc^	2941.23 ± 156.82 ^b^	1487.40 ± 36.865 ^f^	2482.32 ± 136.75 ^d^	2519.96 ± 163.76 ^cd^
4-*O*-caffeoylquinic acid	981.08 ± 63.18 ^bcd^	869.65 ± 48.95 ^e^	1365.16 ± 89.10 ^a^	882.50 ± 57.89 ^de^	1018.33 ± 87.85 ^b^	990.22 ± 64.72 ^bc^	512.62 ± 37.16 ^f^	916.21 ± 66.84 ^ce^	940.69 ± 67.09 ^be^
TMAC	5185.89 ± 89.28 ^bc^	4296.62 ± 199.25 ^e^	7402.83 ± 196.27 ^a^	4205.75 ± 152.57 ^e^	5419.29 ± 211.27 ^bc^	5521.29 ± 195.845 ^b^	2673.11 ± 52.17 ^f^	4802.21 ± 157.28 ^d^	5021.03 ± 211.27 ^cd^
3,4-di-*O*-caffeoylquinic acid	718.39 ± 39.28 ^bc^	517.04 ± 15.73 ^d^	782.14 ± 23.18 ^a^	498.48 ± 38.93 ^d^	743.78 ± 28.33 ^ab^	719.32 ± 47.38 ^bc^	229.17 ± 17.03 ^e^	677.70 ± 13.98 ^c^	685.30 ± 20.17 ^c^
3,5-di-*O*-caffeoylquinic acid	7156.41 ± 301.27 ^b^	5913.21 ± 392.83 ^c^	8119.70 ± 503.27 ^a^	7090.86 ± 411.28 ^b^	8259.63 ± 367.86 ^a^	7305.74 ± 522.86 ^b^	4489.29 ± 289.78 ^d^	7251.88 ± 298.39 ^b^	6717.60 ± 363.18 ^b^
4,5-di-*O*-caffeoylquinic acid	4820.02 ± 110.37 ^ab^	4217.64 ± 207.81 ^b^	4520.06 ± 167.28 ^ab^	4360.67 ± 112.13 ^ab^	4956.98 ± 165.32 ^a^	4791.11 ± 226.83 ^ab^	3000.33 ± 162.18 ^cd^	3250.84 ± 255.35 ^c^	4551.21 ± 182.86 ^ab^
TDAC	12694.82 ± 398.37 ^bc^	10647.89 ± 458.76 ^e^	13421.90 ± 637.81 ^ab^	11950.01 ± 502.85 ^cd^	13960.39 ± 411.15 ^a^	12816.17 ± 628.75 ^bc^	7718.79 ± 327.27 ^f^	12515.29 ± 428.63 ^de^	11998.11 ± 463.37 ^cd^
caffeic acid	-	4.29 ± 0.11 ^f^	23.03 ± 0.98 ^a^	7.43 ± 0.26 ^e^	11.39 ± 0.28 ^c^	13.50 ± 0.09 ^b^	13.40 ± 0.36 ^b^	4.70 ± 0.12 ^f^	9.92 ± 0.81 ^d^
TPAC	17880.71 ± 411.8 ^c^	14948.80 ± 583.67 ^e^	20847.76 ± 762.17 ^a^	16163.18 ± 598.17 ^de^	19391.07 ± 512.28 ^b^	18350.97 ± 703.84 ^bc^	10405.31 ± 356.83 ^f^	17322.21 ± 501.25 ^de^	17029.06 ± 581.25 ^cd^
hyperoside	919.83 ± 38.19 ^ab^	875.45 ± 58.91 ^bcd^	994.81 ± 67.73 ^a^	865.66 ± 33.83 ^bcd^	797.63 ± 68.39 ^d^	919.97 ± 57.38 ^abc^	708.80 ± 46.86 ^e^	860.22 ± 50.09 ^bcd^	875.19 ± 58.96 ^bcd^
luteoloside	1939.73 ± 60.8 ^b^	1878.94 ± 97.95 ^bc^	2331.10 ± 96.06 ^a^	1775.10 ± 72.81 ^bcd^	1711.19 ± 87.27 ^cd^	1881.30 ± 99.96 ^b^	1614.81 ± 118.19 ^d^	1923.38 ± 148.71 ^b^	2416.07 ± 99.26 ^a^
apigenin-7-*O*-glucoside	2651.70 ± 56.38 ^cd^	2461.46 ± 162.84 ^def^	1553.48 ± 98.98 ^h^	2493.06 ± 83.87 ^cf^	2143.65 ± 48.78 ^g^	2522.15 ± 138.39 ^cde^	2905.07 ± 57.81 ^b^	2662.34 ± 187.29 ^c^	3539.55 ± 163.83 ^a^
linarin	2816.67 ± 67.38 ^cd^	2623.92 ± 162.18 ^def^	3470.66 ± 207.48 ^ab^	2795.03 ± 156.27 ^ce^	3239.20 ± 187.53 ^b^	3573.51 ± 165.78 ^a^	2518.28 ± 89.96 ^f^	2726.28 ± 127.28 ^cf^	2898.40 ± 120.53 ^c^
luteolin	40.11 ± 1.99 ^e^	52.47 ± 1.08 ^c^	70.68 ± 2.83 ^a^	55.18 ± 1.87 ^b^	46.99 ± 0.99 ^d^	24.08 ± 0.57 ^f^	41.32 ± 1.28 ^e^	51.55 ± 0.98 ^c^	53.15 ± 2.55 ^bc^
apigenin	-	94.36 ± 2.76 ^a^	43.29 ± 0.27 ^b^	8.90 ± 0.26 ^e^	17.83 ± 1.01 ^d^	10.67 ± 0.72 ^e^	23.88 ± 0.92 ^c^	-	9.65 ± 0.39 ^e^
acacetin	-	-	-	-	-	-	-	-	-
TFC	8368.04 ± 98.25 ^bc^	7986.60 ± 212.38 ^c^	8464.01 ± 198.26 ^bc^	7992.93 ± 178.84 ^c^	7956.49 ± 223.17 ^c^	8931.68 ± 256.27 ^b^	7812.16 ± 172.38 ^c^	8223.77 ± 167.83 ^c^	9792.01 ± 187.22 ^a^
TKPC	11745.82 ± 421.72 ^cd^	9973.12 ± 472.46 ^e^	13144.12 ± 652.89 ^a^	11071.71 ± 541.23 ^d^	12730.64 ± 328.48 ^ab^	12128.27 ± 331.34 ^bc^	7591.50 ± 198.62 ^f^	11657.58 ± 348.80 ^cd^	11653.63 ± 351.28 ^cd^

Data are the mean ± standard deviation of triplicate independent experiments. “-” represents ‘not detected’. ^a, b, c, d, e, f, g, h^ Different letters in each raw indicate significant difference at a significant level of 0.05. TMAC: Total mono-caffeoylquinic acid contents, TDAC: Total di-caffeoylquinic acid contents, TPAC: Total phenolic acid contents, TFC: Total flavonoids contents, TKPC: Three key phenolic components contents.

**Table 3:** Antioxidant activities in different ‘Duoju’.

Antioxidant Capacity	DJ1	DJ2	DJ3	DJ4	DJ5	DJ6
DPPH *	2.47 ± 0.05 ^c^	1.75 ± 0.04 ^f^	3.04 ± 0.08 ^a^	2.68 ± 0.03 ^b^	2.10 ± 0.08 ^e^	2.23 ± 0.04 ^d^
ABTS *	2.77 ± 0.06 ^ab^	2.30 ± 0.05 ^d^	2.83 ± 0.04 ^a^	2.71 ± 0.03 ^b^	2.13 ± 0.06 ^e^	2.39 ± 0.03 ^c^
FRAP *	222.28 ± 4.10 ^d^	247.73 ± 8.45 ^b^	236.82 ± 3.19 ^c^	255.00 ± 8.20 ^b^	266.82 ± 4.16 ^a^	273.64 ± 7.10 ^a^

Data are the mean ± standard deviation of triplicate independent experiments. *: The unit of EC_50_ in ABTS and DPPH assays is mg/mL, whereas the unit for FRAP analysis is mg TEAC/g DW. ^a, b, c, d, e, f^ Different letters in each raw indicate a significant difference at a significant level of 0.05.

**Table 4:** Antioxidant activities in different ‘Taiju’.

Antioxidant Capacity	TJ1	TJ2	TJ3	TJ4	TJ5	TJ6	TJ7	TJ8	TJ9
DPPH *	1.74 ± 0.06 ^e^	2.13 ± 0.06 ^b^	2.05 ± 0.10 ^bc^	1.70 ± 0.7 ^e^	1.93 ± 0.10 ^cd^	1.69 ± 0.04 ^e^	2.56 ± 0.20 ^a^	1.78 ± 0.07 ^de^	1.97 ± 0.09 ^bc^
ABTS *	1.84 ± 0.07 ^de^	2.09 ± 0.06 ^b^	1.94 ± 0.10 ^cd^	1.82 ± 0.03 ^ef^	1.88 ± 0.08 ^cde^	1.91 ± 0.04 ^cde^	2.42 ± 0.06 ^a^	1.88 ± 0.07 ^cde^	1.98 ± 0.08 ^bc^
FRAP *	387.73 ± 3.28 ^b^	390.15 ± 2.78 ^b^	362.87 ± 8.45 ^cd^	362.88 ± 9.72 ^cd^	352.88 ± 1.89 ^d^	368.94 ± 4.30 ^c^	320.15 ± 4.58 ^e^	436.51 ± 5.91 ^a^	318.93 ± 8.25 ^e^

Data are the mean ± standard deviation of triplicate independent experiments. *: The unit of EC_50_ in ABTS and DPPH assays is mg/mL, whereas the unit for FRAP analysis is mg TEAC/g DW. ^a, b, c, d, e, f^ Different letters in each raw indicate the significant difference at a significant level of 0.05.

**Table 5:** Antioxidant activities of individual phenolic compounds.

Phenolic Compound	DPPH (EC_50_ μmol/L)	ABTS (EC_50_ μmol/L)	FRAP (mg TEAC/g DW)
3-*O*-caffeoylquinic acid	22.81 ± 0.22 ^de^	18.41 ± 0.43 ^d^	255.92 ± 13.29 ^de^
5-*O*-caffeoylquinic acid	17.62 ± 0.33 ^ef^	10.27 ± 0.21 ^de^	366.00 ± 21.13 ^a^
4-*O*-caffeoylquinic acid	20.30 ± 0.20 ^de^	12.52 ± 0.10 ^de^	273.25 ± 14.30 ^cd^
caffeic acid	16.73 ± 0.31 ^ef^	11.57 ± 0.18 ^de^	224.23 ± 16.56 ^gh^
hyperoside	8.44 ± 0.10 ^ef^	14.01 ± 0.37 ^de^	235.00 ± 0.10 ^fg^
luteoloside	9.99 ± 0.42 ^ef^	14.22 ± 0.65 ^de^	212.17 ± 0.10 ^h^
3,4-di-*O*-caffeoylquinic acid	10.43 ± 0.33 ^ef^	6.14 ± 0.33 ^e^	297.02 ± 9.18 ^b^
3,5-di-*O*-caffeoylquinic acid	10.48 ± 0.22 ^ef^	7.14 ± 0.47 ^e^	249.94 ± 25.16 ^ef^
apigenin-7-*O*-glucoside	307.44 ± 13.10 ^b^	158.24 ± 6.22 ^b^	53.87 ± 3.89 ^j^
4,5-di-*O*-caffeoylquinic acid	9.40 ± 0.31 ^ef^	5.47 ± 0.43 ^e^	279.09 ± 15.80 ^bc^
linarin	-	256.03 ± 10.18 ^a^	6.56 ± 0.34 ^k^
luteolin	9.81 ± 0.45 ^ef^	8.06 ± 0.50 ^e^	229.93 ± 5.87 ^g^
apigenin	199.38 ± 19.11 ^c^	144.55 ± 7.36 ^c^	54.62 ± 1.98 ^j^
acacetin	706.27 ± 35.90 ^a^	254.17 ± 16.75 ^a^	51.08 ± 1.20 ^j^
*Positive Control* (*Vitamin C*)	34.69 ± 1.47 ^d^	11.77 ± 0.33 ^de^	168.93 ± 8.30^i^

Data are the mean ± standard deviation of triplicate independent experiments. “-” represents ‘not detected’. ^a, b, c, d, e, f, g, h, i, j, k^ Different letters in each raw indicate significant difference at a significant level of 0.05.

**Table 6:** Correlation index (*R*^2^) between phenolic compounds and antioxidant activities in different ‘Hangbaiju’.

Phenolic Compound	DPPH		ABTS		FRAP	
	Duoju	Taiju	Duoju	Taiju	Duoju	Taiju
5-*O*-caffeoylquinic acid	0.8231	0.5808	0.7966	0.6304	0.2733	0.5166
3,5-di-*O*-caffeoylquinic acid	0.7134	0.5761	0.6871	0.6147	0.0831	0.4985
luteoloside	0.6063	0.5687	0.5455	0.6339	0.0369	0.1208
TMAC	0.8101	0.5279	0.6871	0.5704	0.3318	0.6051
TDAC	0.8433	0.5917	0.7982	0.6599	0.2568	0.4081
TPAC	0.8655	0.5886	0.8118	0.6480	0.2686	0.4987
TFC	0.7165	0.5810	0.6686	0.6522	0.0245	0.0835
TKPC	0.8761	0.5963	0.8224	0.6602	0.2139	0.4265

TMAC: Total mono-caffeoylquinic acid contents, TDAC: Total di-caffeoylquinic acid contents, TPAC: Total phenolic acid contents, TFC: Total flavonoids contents, TKPC: Three key phenolic components contents.

## References

[1] Chen L., Kotani A., Kusu F., Wang Z., Zhu J., Hakamata H. (2015). Quantitative comparison of caffeoylquinic acids and flavonoids in *Chrysanthemum morifolium* flowers and their sulfur-fumigated products by three-channel liquid chromatography with electrochemical detection. Chem. Pharm. Bull..

[2] Qu L., Ruan J.Y., Jin L.J., Shi W.Z., Li X.X., Han L.F., Zhang Y., Wang T. (2017). Xanthine oxidase inhibitory effects of the constituents of *Chrysanthemum morifolium* stems. Phytochem. Lett..

[3] Wang S., Hao L.J., Zhu J.J., Zhang Q.W., Wang Z.M., Zhang X., Song X. (2014). Study on the effects of sulfur fumigation on chemical constituents and antioxidant activity of *Chrysanthemum morifolium* cv. Hang-ju. Phytomedicine.

[4] Ma D., Wako Y. (2017). Evaluation of phenolic compounds and neurotrophic/neuroprotective activity of cultivar extracts derived from *Chrysanthemum morifolium* flowers. Food Sci. Technol. Res..

[5] Lin L.Z., Harnly J.M. (2010). Identification of the phenolic components of chrysanthemum flower (*Chrysanthemum morifolium* Ramat.). Food Chem..

[6] Wang T., Guo Q.S., Mao P.F. (2014). Flavonoid accumulation during florescence in three *Chrysanthemum morifolium* ramat cv. ‘Hangju’ genotypes. Biochem. Syst. Ecol..

[7] Chinese Pharmacopoeia Committee (2015). Pharmacopoeia of the People’s Republic of China.

[8] He D.X., Ru X.C., Wen L., Wen Y.C., Jiang H.D., Bruce I.C., Jin J., Ma X., Xia Q. (2012). Total flavonoids of Flos Chrysanthemi protect arterial endothelial cells againstoxidative stress. J. Ethnopharmacol..

[9] Zhang N., He Z., He S., Jing P. (2019). Insights into the importance of dietary chrysanthemum flower (*Chrysanthemum morifolium* cv. Hangju)-wolfberry (*Lycium barbarum* fruit) combination in antioxidant and anti-inflammatory properties. Food Res. Int..

[10] Xie Y.Y., Qu J.L., Wang Q.L., Wang Y., Yoshikawa M., Yuan D. (2012). Comparative evaluation of cultivars of *Chrysanthemum morifolium* flowers by HPLC-DAD-ESI/MS analysis and antiallergic assay. J. Agric. Food Chem..

[11] Jiang Y., Ning Z., Li S. (2018). Extraction and purification of isochlorogenic acid c from *Chrysanthemum morifolium* using ionic liquid-based ultrasound-assisted extraction and aqueous two-phase system. Food Sci. Nutr..

[12] Li Y., Yang P., Luo Y., Gao B., Sun J., Lu W., Liu J., Chen P., Zhang Y., Yu L. (2019). Chemical compositions of chrysanthemum teas and their anti-inflammatory and antioxidant properties. Food Chem..

[13] Yuan J., Hao L.J., Wu G., Wang S., Duan J.A., Xie G.Y., Qin M.J. (2015). Effects of drying methods on the phytochemicals contents and antioxidant properties of *Chrysanthemum* flower heads harvested at two developmental stages. J. Funct. Foods.

[14] Shi J.Y., Gong J.Y., Liu J.E., Wu X.Q., Zhang Y. (2009). Antioxidant capacity of extract from edible flowers of *Prunus mume* in China and its active components. LWT Food Sci. Technol..

[15] Hwang S.H., Paek J.H., Lim S.S. (2016). Simultaneous ultra performance liquid chromatography determination and antioxidant activity of linarin, luteolin, chlorogenic acid and apigenin in different parts of compositae species. Molecules.

[16] Shi G., Yang S., Zhang X., Liu J., Liu Z., Zhao Y. (2015). Simultaneous determination of five flavonoids components in different parts of *Callistephus chinensis* by HPLC. Chin. Trad. Herbal Drugs.

[17] Turkoglu A., Duru M.E., Mercan N., Kivrak I., Gezer K. (2007). Antioxidant and antimicrobial activities of *Laetiporus sulphureus* (bull.) murrill. Food Chem..

[18] Re R., Pellegrini N., Proteggente A., Pannala A., Yanga M., Rice-Evansa C. (1999). Antioxidant activity applying an improved ABTS radical cation decolorization assay. Free Radic. Biol. Med..

[19] Benzie I.F.F., Strain J.J. (1996). Uric acid: Friend or foe?. Redox Rep..

[20] Dudonné S., Vitrac X., Coutière P., Woillez M., Mérillon J.M. (2009). Comparative study of antioxidant properties and total phenolic content of 30 plant extracts of industrial interest using DPPH, ABTS, FRAP, SOD, and ORAC assays. J. Agric. Food Chem..

[21] Lin S., Li H.Y., Wang Z.Y., Liu X., Yang Y., Cao Z.W., Du G., Zhao L., Zhang Q., Wu D.T. (2019). Analysis of methanolic extracts and crude polysaccharides from the leaves of *Chuanminshen violaceum* and their antioxidant activities. Antioxidants.

[22] Thaipong K., Boonprakob U., Crosby K., Cisneros-Zevallos L., Byrne D.H. (2012). Comparison of ABTS, DPPH, FRAP, and ORAC assays for estimating antioxidant activity from guava fruit extracts. J. Food Compos. Anal..

[23] Gong J.Y., Xia D.Z., Huang J., Ge Q., Mao J.W., Liu S.W., Zhang Y. (2015). Functional components of bamboo shavings and bamboo leaf extracts and their antioxidant activities in Vitro. J. Med. Food.

[24] Gong J., Huang J., Xiao G., Chen F., Lee B., Ge Q., You Y., Liu S., Zhang Y. (2016). Antioxidant capacities of fractions of bamboo shaving extract and their antioxidant components. Molecules.

[25] Djebbar A., Nassima C., Dina A., Meriem B., Nadjet D., Hania B. (2009). Flavonoids in human health: From structure to biological activity. Curr. Nutr. Food Sci..

[26] Taira J., Uehara M., Tsuchida E., Ohmine W. (2014). Inhibition of the β-catenin/Tcf signaling by caffeoylquinic acids in sweet potato leaf through down regulation of the Tcf-4 transcription. J. Agric. Food Chem..

[27] Šamec D., Maretic’ M., Lugaric’ I., Mešic’ A., Salopek-Sondi B., Duralija B. (2016). Assessment of the differences in the physical, chemical and phytochemical properties of four strawberry cultivars using principal component analysis. Food Chem..

[28] Dawidowicz A.L., Typek R. (2014). Transformation of 5-O-Caffeoylquinic acid in blueberries during high-temperature processing. J. Agric. Food Chem..

[29] Deshpande S., Jaiswal R., Matei M.F., Kuhnert N. (2014). Investigation of acyl migration in mono- and dicaffeoylquinic acids under aqueous basic, aqueous acidic, and dry roasting conditions. J. Agric. Food Chem..

[30] Xue M., Shi H., Zhang J., Liu Q.Q., Guan J., Zhang J.Y., Ma Q. (2016). Stability and degradation of caffeoylquinic acids under different storage conditions studied by high-performance liquid chromatography with photo diode array detection and high-performance liquid chromatography with electrospray ionization collision-induced dissociation tandem mass spectrometry. Molecules.

[31] Nishihara M., Nakatsuka T., Yamamura S. (2005). Flavonoid components and flower color change in transgenic tobacco plants by suppression of chalcone isomerase gene. FEBS Lett..

[32] Bobilya D.J. (2002). Flavonoid antioxidants: Chemistry, metabolism and structure-activity relationships. J. Nutr. Biochem..

[33] Cao G., Sofic E., Prior R.L. (1997). Antioxidant and prooxidant behavior of flavonoids: Structure-activity relationship. Free Radic. Biol. Med..

[34] Benzo F.A. (2017). Antioxidant and antidiabetic effects of flavonoids: A structure-activity relationship based study. BioMed Res. Int..

